# Analysis of 232 total fertilization failure cycles during intracytoplasmic sperm injection

**Published:** 2011

**Authors:** Esma Sarikaya, Ozlem Gun Eryilmaz, Ruya Deveer, Muammer Dogan, Leyla Mollamahmutoglu

**Affiliations:** Centre for Reproductive Medicine, Zekai Tahir Burak Women's Health Research and Education Hospital, Ankara, Turkey.

**Keywords:** *Total fertilization failure*, *ICSI*, *Cyclecharasteristics*

## Abstract

**Background:** The intracytoplasmic sperm injection procedure ending with total fertilization failure is very distressfull event for both the clinician and the patient.

**Objective:** The aim of this study was to identify independent factors which could be used to identify total fertilization failure before the day of intracytoplasmic sperm injection.

**Materials and Methods: **This was a retrospective study of 232 patients who were admitted to a tertiary-care hospital IVF Unit and showed total fertilization failure during intracytoplasmic sperm injection cycles. To sort out the interwined effects of female age, basal FSH, sperm quality, antral follicle count, starting dose of gonadotrophine, sperm extraction technique, cycle length, >14 mm follicle number, oocyte number after oocyte pick up, estradiol and progesterone level on the day of hCG and the MI, MII and GV oocyte number on the fertilization, multiple logistic regression analysis was used.

**Results: **The total fertilization failure rate was 6% and the recurrance rate was 23%. The original model illustrated that the presence of GV oocytes, total oocyte number less than six, <2000 pg/mL E2 concentration on the day of hCG and testicular sperm extraction increases the total fertilization failure risk.

**Conclusion**: It is very difficult to predict total fertilization failure. Sometimes even with one good quality oocyte and sperm and in the case of globozoospermia fertilization can be achieved. Not only azoospermia but also low oocyte numbers increase the chance of total fertilization failure even after intracytoplasmic sperm injection.

## Introduction

Intracytoplasmic sperm injection (ICSI) give the chance to the couples with severe male factor infertility to achieve fertilization and clinical pregnancy rates comparable to other in-vitro fertilization (IVF) patients. 

ICSI bypasses all physiologic sperm screening mechanisms and leads to fertilization with microsurgical epididiymal sperm aspiration (MESA), testicular sperm exicision (TESE) and even round spermatid nuclear injection (ROSNI), with results similar to those of ejaculated sperm. However, total fertilization failure (TFF) still occurs in some patients despite the utilization of ICSI (1).

The term TFF refers to failure of all the available oocytes to be fertilized and it is very distressfull event for both the clinician and the patient. It occurs in 1-10% of IVF cycles and 2-3% of ICSI cycles (1, 2). Taylor *et al* found no difference between the incidence of TFF following IVF (4.0%) and ICSI (4.5%) (3,4). Recurrence of TFF in subsequent IVF cycles is approximately 30%. ICSI was initially utilized to overcome TFF. Despite the prevalent use of ICSI, the fertilization rate remains around 50–70%, which appears to be no different than standard IVF with non-male factor. This suggests that factors other than sperm binding and penetration may limit the fertilization rate. 

Operator experience is undoubtedly important for the ICSI success, but other published reasons for TFF are: total immotility of spermatozoa, teratozoo-spermia, low oocyte yield, oocyte aneuploidy, fragile oocytes, defects in the in-vitro sperm/ oocyte medium and ICSI technique (5). A small number of couples will have TFF for no apparent reason-they have egg and sperm with apparently normal or near-normal morphology leaving no obvious reason for the failure. In such cases the primary reason for TFF after ICSI appears to be lack of oocyte activation (1, 6-9). Our aim was to examine the chance of prediction of the TFF before the day of ICSI with known semen and cycle characteristics.

## Materials and methods

This study was approved by the local Ethics Committee of Dr. Zekai Tahir Burak Women Health and Research Hospital**. **The files of the couples who showed TFF during ICSI cycles between March 2002 and September 2007 were retrospectively examined in Zekai Tahir Burak Women's Health Research and Education Hospital IVF Unit, Ankara, Turkey. A total of 232 TFF cycles and 205 control groups in which fertilization were achieved included in this retrospective, single-institution, cross-sectional analysis. 

**Inclusion criteria**

Patients, who were at the primary infertility age, (20-38 years) were included. All patients had no hormonal or non-hormonal therapy for the last 3 months and had no systemic illness. Patients with FSH>13 were excluded from the study. In no instance was donor sperm or oocyte used for ICSI since it is forbidden in law in Turkey. 

**Laboratory methods**

Ovarian stimulation in most patients consisted of mostly GnRH agonist down-regulation, followed by rec FSH/hMG, but microdose flare, or antagonist protocols were also used. Follicular development was monitored and when the maximal diameter of three leading follicles exceeded 18 mm, 10000 IU hCG (Pregnyl 5000 IU Amp. Organon) was given intramuscularly. After 36 hours, follicles were aspirated under transvaginal ultrasound guidance with a single lumen aspiration needle. 2 hours post-collection, the oocytes were denuded of their surrounding cumulus cells with hyaluronidase (Vitrolife, Sweden) and mechanical pipeting, which allows a precise determination of nuclear maturation status and oocyte morphology.

**Sperm analysis and sperm processing**

The semen samples were collected on the day of oocyte pick up by masturbation after 3-4 days of abstinance. Routine semen analysis was done by light microscopy according to strict criteria. Morphology was evaluated by Papanicolau staining technique. 

Density gradient centrifugation of spermatazoa from fresh human ejaculates was done by PureSperm Wash (NidaCon International AB PSW-100). Testicular biopsies were performed under local anaesthesia, one day before oocyte pick up. Repeated sampling was performed in some cases in order to find sperm and when necessary contralateral testis was also biopsied. 

**Intracytoplasmis sperm injection**

The best available motile and morphologically well shaped spermatozoon was aspirated from the separate sperm droplet into the injection pipette and then transferred to the 10% PVP droplet and attaching cells and debris is seperated. If there were only completely immotile sperm cells, spermatozoa with normal morphology were selected by objective morphometry and Kruger strict morphology for the microinjection procedure. Sperm viability tests and recently introduced sperm selection techniques (2) was not relevant at that time in our laboratory. 

Both motile and immotile spermatozoa were immobilized by touching its tail near the midpiece with the injection pipette and aspirated into the injection pipette, tail first. Only mature oocytes (metaphase II) were injected according to the methodology described by Van Steirteghem *et al* (10). 

The oocytes were examined immediately after the injection procedure to evaluate the placement of the sperm and the intactness of the oolemma. After the injection prosedure, the oocytes were washed in fresh media and incubated in G1, 3 medium (vitrolife, Sweden). 

The injected oocytes were examined for survival and normal fertilization (the presence of pronuclei) 18 hr after ICSI under an inverted microscope (Zeiss, Germany). Routinely a fertilization recheck was performed 4-6 hr later to identify any delayed fertilizations. ICSI was performed by the 2 experienced embryologist and the same technique and culture condition was used. None of the TFF cycles during this time were the result of equipment or technical problems; therefore, the failures could be attributed to patient variability.

**Statistical analysis**

The data were analyzed with SPSS 11.5 package program. Continuous variables were expressed as mean±standard deviation or median (minimum-maximum), where applicable. Qualitative data were presented as number of patients and percentage (%). Univariate analysis was done for each variable to assess distribution and the need for transformation. Differences between groups were analyzed using independent samples t-test for continuous data, whilst χ^2 ^was used for categorical data. The model was developed using logistic regression with the total fertilization failure as the outcome of interest. For multiple logistic analysis, significant risk factors were selected by using the stepwise selection method. Odds ratio and 95% CIs for each independent variables were calculated. p<0.05 was considered statistically significant. 

## Results

The overall fertilization rate (2PN/MII oocyte) in these 3862 ICSI cycles from March 2002, through September 2007, was 74% of intact oocytes. However, in 232 of these cycles, none of the injected oocytes became fertilized, so the TFF rate was 6%. The mean female age (±SD) of this group was 31.0±5.61 and mean male age was 34.1±5.91years. Infertility causes among patients with TFF are shown in table I. Demographic, reproductive and cycle characteristics of TFF(+) and (-) group are shown in table II. Eighty four couples tried one, 25 couples tried two and 4 couples tried three more ICSI cycle (total 113 couples).

Of 113 couples, 67 (57%) achieved fertilization in their subsequent ICSI cycles. 20 of 113 couples’ cycle was cancelled (17%) mostly due to: insufficient ovarian response (15), no sperm from TESE (2) and OHSS risk (1). TFF recurrance rate was 23% in subsequent ICSI cycle. TFF has repeated 2 times in one couple and 3 times in another. In both of them spermatozoa had extremely amorphous head, with no acrosome.

To sort out the interwined effects of; female age, basal FSH level, sperm quality, antral follicle count, starting dose of gonadotrophine, sperm extraction technique, cycle length, >14 mm follicle number, oocyte number after OPU, E2 and progesteron level on the day of hCG and MI, MII, GV oocyte number on the fertilization multiple logistic regression backward LR procedure was applied. 

The original model illustrated that the presence of GV oocytes (OR 1.53 95% CI 1.26-1.87: p<0.001), total oocyte number less than six (OR3.73 95% CI 1.93-7.19 p<0.001) <2000 pg/mL E2 concentration on the day of hCG (OR1.53 95% CI 1.04-2.57: p=0.002) and cycles in which sperm was retrieved from TESE (OR 2.92 1.67-5.10% p<0.001 ) increases the TFF risk (Table III).

**Table I:** Indications for ICSI in TFF (+) group

**ICSI indication**	**Number of patients**
Azoospermia	126
Unexplained infertility	75
More than one reason	17
Advanced female age	14

**Table II:** Demographic, reproductive and cycle characteristics of TFF(+) and TFF(-) group

		**TFF(-) (n=205)**	**TFF(+) (n=232)**	**p-value**
Female age (years)		30.3±5.61	31.0±5.61	NS
Male age (years)		34.5±5.47	34.1±5.91	NS
Duration of infertility (years)		10.0±5.16	10.0±5.29	NS
Antral follicle count				
	<4	47 (%23.2)	64 (%27.6)	NS
	5-10	93 (%45.8)	119 (%51.3)	
	>10	63 (%31.0)	49 (%21.1)	
Basal FSH (IU/L)				
	≤10	193 (%94.1)	219 (%94.4)	NS
	>10	12 (%5.9)	13 (%5.6)	
Protocol				
	Long Lucrin	162 (%79.0)	181 (%78.0)	NS
	Microdose and antag	20 (%9.8)	30 (%12.9)	
	Others	23 (%11.2)	21 (%9.1)	
Drug used				
	Only rec FSH	78 (%38.0)	87 (%37.5)	NS
	RecFSH+rec LH	17 (%8.3)	33 (%14.2)	
	RecFSH+hMG	85 (%41.5)	86 (%37.1)	
	Only hMG	25 (%12.2)	26 (%11.2)	
P (ng/mL) on the day of hCG				
	≤0.9	127 (%62.0)	151 (%65.1)	NS
	>1	78 (%38.0)	81 (%34.9)	
E2 (pg/mL) on the day of hCG				
	<500	7 (%3)	12 (%5,1)	0.023
	500-1000	22 (%10.7)	29 (%12.5)	
	1001-2000	58 (%28.3)	99 (%42.7)^†^	
	2001-3000	63 (%30.7)	53 (%22.8)	
	3001-4000	26 (%12.7)	20 (%8.6)	
	>4000	24 (%11.7)	24 (%10.3)	
Retrieved oocyte number				
	<3	37 (%18.0)	51 (%22.0)	0.002
	3-6	38 (%18.5)	72 (%31.0)^†^	
	6-10	73 (%35.6)	70 (%30.2)	
	>10	57 (%27.8)	39 (%16.8)^†^	
Oocyte maturity				
	M1	1 (0 – 11)	1 (0 – 15)	0.028
	M2	5 (0 – 20)	3 (0 – 16)	<0.001
	GV	0 (0 – 6)	0 (0 – 11)	<0.001
Cycle length (day)				
	<8	26 (%12.7)	17 (%7.3)	NS
	8-11	139 (%67.8)	163 (%70.3)	
	>11 +	40 (%19.5)	52 (%22.4)	
Coasting (day)				
	0	176 (85.9%)	205 (88%)	NS
	1	16 (7.8%)	12 (%5.2)	
	2	16 (7.8%)	12 (%5.2)	
	3	7 (3.4%)	6 (2.6%)	
	5	1 (0.5%)	0 (0%)	
Rapidly progressive motil sperm				
	<5%	129 (%62.9)	141 (%60.8)	NS
	5-15%	38 (%18.5)	35 (%15.1)	
	16-30%	29 (%14.1)	32 (%13.8)	
	>30%	9 (%4.4)	24 (%10.3)	
Kruger				
	<4%	131 (%63.9)	132 (%56.9)	NS
	4-8%	25 (%12.2)	44 (%19.0)	
	>8%	49 (%23.9)	56 (%24.1)	

Values are given as n (%), median (ranges) , or mean (±SD). NS, not significant.

**Table III:** Multiple logistic regression analysis with the total fertilization failure as the outcome of interest

**Independent variables**		**p-value**	**OR**	**(95%CI)**
Retrieved oocyte number				
	<3	0.005	2.71	( 1.34-5.46)
	3-6	<0.001	3.73	(1.93- 7.19)
Oocyte maturity (GV)		<0.001	1.53	(1.26-1.87)
E2 (≤2000pg/ml)		0.032	1.64	(1.04-2.57)
Sperm origin (TESE)		<0.001	2.92	(1.67-5.10)

## Discussion

It is important to properly select candidates for ICSI before the procedure. It is not cost effective to demand that all infertile couples first demonstrate TFF, using standart IVF. Appropriate markers are necessary to predict TFF and counsel patient. To identify and evaluate the statistically significant predictors of ICSI fertilization rates, Shen et al. developed a statistical model and found that; sperm motility and ICSI operator are the two most important predictors for the ICSI fertilization rate in vitro (11). 

Low number of oocytes after oocyte pick up, damage of oocytes after ICSI, fragile and morphologically abnormal oocytes in poor responders are the reasons of oocyte borne activation failure (12). As eggs age, nondisjunctional events at the time of ovulation occur more frequently and such events at the first meiotic division is the most commen cause of failure of assisted reproduction techniques (13,14). 

Current trends to improve pregnancy rates and reduce the risk of multiple pregnancy and OHSS, have created a dilemma. The question of what reasonable number of oocytes needed? arises. The fewer the oocytes obtained, the higher the risk of all of them being immature. ICSI is performed only on mature MII oocytes. Furthermore, because fertilization rates are not 100% even in ICSI, the risk of TFF can be reduced by injection of an appropriate number of oocytes(15). Flaherty *et al* found that TFF rate was 37% when only one oocyte injected, this rate was 13% and 0.8% with the injection of two and five and more oocytes respectively. 

Melie *et al* reported 11.8% chance of TFF when average number of 2.2 oocytes injected. They concluded reasonable number as 6-10 oocyte (5, 16). Our results concure with the mentioned studies above. Egg number and probably egg quality now emerge as the critical determinants of success for infertility cases involving severe male infertility. During IVF, sperm quality and number of oocytes are important for the prediction of TFF. There are three studies that give two different sperm parameters for deciding ICSI or IVF. Johann et al. concluded that low responders (<4 follicles) needed a postwash total progressive motile sperm count (TPMC) of >2.2×10^6 ^cells to reduce the risk of TFF to <25%. High responders (>15 follicles) needed only 0.35×10^6 ^TPMC. When postwash TPMC and number of follicles are known and an unacceptable TFF outcome is expected, they propose an ICSI procedure a few days before the day of ovum pick up (17).Gozlan *et al* reported that in poorly responding patients, semen quality should remain the most important determinant when considering whether to perform ICSI. They have found that the values of 20×10^6^/mL and 35% motility are good predictors of success in such patients (18). 

Repping *et al* proposed a model at the time of oocyte pick up, including the number of oocytes, both pre- and postwash TMC, which allows an accurate prediction of the chance of TFF and is useful in counseling patients (19). But in ICSI cycles even in poor responders we have found no relation with sperm total motility and morphology and TFF. Even though sperm morphology is still considered to be the best predictor of fertilization for the natural method, the conventional IVF and the intrauterine insemination, many studies have found no correlation between sperm morphology and success with ICSI (22). According to previous reports and our results in ICSI, fertilization may be achieved, even in the peresence of a few motile sperm and globozoospermia because naturel selection steps are skipped in ICSI. Only one condition had a strongly negative influence on the result of ICSI: where an immotile (presumably dead) spermatozoon was injected into the oocyte. Thus for successful ICSI the only ultimate criterion is the presence of at least one motile spermatozoa per oocyte. In most studies, it is found that the fertilizability of totally immotile ejaculated spermatozoa was significantly lower when compared with the fertility capacity of initially immotile but further motile sperm and proposed usage of testicular motile spermatozoa in these cases (20-26) .

The sperm origin may be a limiting factor for fertilization during ICSI. Many studies compared the fertilization rates in ICSI, using sperm from ejaculates of normal and abnormal semen, epididymal sperm, and testicular sperm of obstructive and nonobstructive azoospermic patients. They have found that; the fertilizing ability of sperm in ICSI is highest, with normal semen and lowest with sperm extracted from a testicular biopsy in nonobstructive azoospermia. There was no significant difference in fertilization rates and PRs in the study of Aboulghar *et al* But Göker *et al *have found that the clinical PRs are significantly lower in ICSI with sperm from testicular biopsy (27, 28). Our data concur with that of previous studies suggesting the rate of TFF is higher in cycles in which sperm was retrieved from testicular biopsies in general. However Hammadeh *et al *found no difference in fertilization rate in patients with ejaculated sperms or sperms obtained from testicular extracts (29)**. **

E2 drops and elevated progesterone levels generally thought to be result of poor cycle control**.** Urman *et al *showed no adverse effect of high serum progesterone levels on the day of hCG administration on implantation rates after ICSI and ET (30). Our results concure with these studies. We have found that cycle length and coasting day duration has no effect on TFF rate. 

Strengths and limitations

The validity of our study is strengthened by the inclusion of a relatively large number of consecutive patients with TFF. In terms of weaknesses, the cross-sectional design of the study implies that we cannot establish a cause-effect relationship. Finally, we cannot exclude the possibility that referral of patients to a tertiary care center might have introduced a selection bias.

In summary, it is very difficult to predict TFF. The history of a previously cancelled ICSI cycle due to poor follicular response or cycle resulted with TFF, could alert us about TFF. We should inform the patient about 23% chance of TFF recurrence risk or 17% cycle cancellation risk in subsequent ICSI cycle. Repeated ICSI treatment in couples with TFF may be useful or necessary. In our study we have found that 57% achieved fertilization in their subsequent ICSI cycles. 

Sperm viability tests (dual-color flow cytometry after propidium iodide, rhodamine 123 (R123), ethidium bromide, carboxyfluorescein diacetate (CFDA) staining, diode laser at the tail), assesment of protamine deficiency with chromomycine A3 (CMA3) staining, hyaluronic acid (HA) or zona pellucida-selected spermatozoa techniques, bisbenzimide, Hoechst 33342, SYBR-14 stains for assessing sperm DNA content and viability, gelatine slide test, polarizing microscope, mouse oocyte activation test (MOAT), motile sperm organeller morphology examination (MSOME), intracytoplasmic morphologically selected sperm injection (IMSI), magnetic cell sorting (MACS), removing the outer plasma membrane and acrosome cap from the spermatozoa before injection, removal of cumulus-corona cells from the injection pole with laser beam and artificial oocyte activation using Calcium ionophore or sperm-specific cytosolic phospholipase C(PLCz) are recently introduced techniques that most current research being conducted to improve the ICSI success(1, 2, 4).

It is essential to find the reasons of the previous TFF in order to prevent recurrence. After that, we can counsel the couple to perform their next ICSI attempt in centers that are experienced in such techniques. If retrieved oocyte number is below 6, and most of them are immature (MI, GV), if E2 level on the day of hCG is below 2000 pg/ml and sperm origin is from TESE, we should inform patient about TFF risk. We declare that we have no conflict of interest.
